# Vaginal Intraepithelial Neoplasia (VaIN) after Hysterectomy Is Strongly Associated with Persistent HR-HPV Infection

**DOI:** 10.3390/cancers16142524

**Published:** 2024-07-12

**Authors:** Maria Teresa Bruno, Marco Marzio Panella, Gaetano Valenti, Salvatore Di Grazia, Francesco Sgalambro, Jessica Farina, Miriam Previti, Liliana Mereu

**Affiliations:** 1 Department of General Surgery and Medical-Surgical Specialty, Gynecology and Obstetrics Unit, Rodolico University Hospital, University of Catania, 95123 Catania, Italy; mpanella@unict.it (M.M.P.); sal.digrazia@policlinico.unict.it (S.D.G.); f.sgalambro@policlinico.unict.it (F.S.); uni303932@studium.unict.it (M.P.); liliana.mereu@unict.it (L.M.); 2 Multidisciplinary Research Center in Papillomavirus Pathology, Chirmed, University of Catania, 95123 Catania, Italy; gaetano.valenti@humanitascatania.it; 3 Gynaecologic Oncology Unit, Humanitas Hospital, 95126 Catania, Italy; 4 Anatomic Pathology Unit, Department of Medical Surgical Sciences and Advanced Technologies “G.F. Ingrassia”, University of Catania, Via Santa Sofia 78, 95123 Catania, Italy; jessica.farina@studium.unict.it

**Keywords:** high-grade VaIN, CIN3, hysterectomy, HPV 16, genotyping, vaginal carcinoma

## Abstract

**Simple Summary:**

VaIN after hysterectomy is a rare intraepithelial neoplasia strongly associated with HR-HPV infection. It is hypothesized that the laparoscopic hysterectomy procedure may cause a higher incidence of VaIN in hysterectomized women. In a retrospective study of 170 women hysterectomized due to CIN3 or due to benign uterine pathology, all cases of high-grade VaIN occurred in women with persistent HPV. The most frequent genotype was 16. All these elements suggest that it is a history of HPV-related disease of the lower genital tract and viral persistence, rather than hysterectomy itself, that should be considered risk factors for the development of high-grade VaIN. A vaginal wall biopsy under colposcopy is recommended to be routinely performed before surgery for patients undergoing hysterectomy due to CIN3 or stage IA cervical cancer, which will help determine the necessary extent of vaginal resection during the procedure.

**Abstract:**

The data from the literature show that women undergoing a LEEP due to CIN3 have a greater risk of having subsequent high-grade anogenital intraepithelial neoplasia or cancer, and the risk is greater for vaginal cancer than for anal and vulvar cancers. It is hypothesized that the laparoscopic hysterectomy procedure may cause a higher incidence of VaIN in hysterectomized women. There are few studies addressing this issue, and they show mixed results. This study aimed to investigate the incidence of high-grade or severe VaIN in the population of women undergoing hysterectomy for CIN3 or benign uterine disease and illustrate the treatment options and follow-up. Methods: This retrospective study was conducted on 170 women who underwent a laparoscopic hysterectomy due to high-grade cervical intraepithelial neoplasia (CIN3) or benign gynecological disease. The follow-up strategy included performing a cotest and colposcopy with biopsy if necessary. The median time between primary treatment and a diagnosis of high-grade VaIN was 18 months. Results: High-grade or severe VaIN was found in eight patients after hysterectomy (4.7%). All cases of high-grade VaIN occurred in women with persistent HPV infection. The most frequent genotype was 16. Women hysterectomized due to CIN3 showed an eight-fold greater risk than women hysterectomized due to benign disease of developing high-grade VaIN. The risk of VaIN is low in women hysterectomized due to benign disease. The risk of developing VaIN is greater in women with viral persistence. Conclusion: All these elements suggest that it is a history of HPV-related disease of the lower genital tract and viral persistence, rather than hysterectomy itself, that should be considered risk factors for the development of high-grade VaIN. After hysterectomy, patients with a history of CIN should undergo annual screening with vaginal dome cytology and HPV testing.

## 1. Introduction

To effectively reduce morbidity and mortality due to cervical cancer, early diagnosis and treatment of grade 3 cervical intraepithelial neoplasia (CIN3) of the uterine cervix are necessary.

CIN3/HSILs represent true preneoplastic lesions of cervical carcinoma; these are lesions typical of young women who are often still nulliparous and eager to have children, so using a therapeutic technique that preserves the functionality of the cervix is of fundamental importance. Excision is the treatment indicated by the American Society Colposcopy and Cervical Pathology (ASCCP), either surgical excision (cold blade conization) or a LEEP (large loop excision procedure) [1]. There is an important difference between the two techniques: surgical conization removes a greater quantity of tissue from the uterine cervix, putting a future pregnancy at risk, so currently, it is preferred to use a LEEP. The problem with LEEP is the fact that there is a high percentage of positive margins and persistence of the viral lesions, which the literature has identified as risk factors for residual disease [2,3,4]. Previous studies have shown that menopausal status also represents a risk factor for residual or occult disease. Cervical atrophy secondary to a lack of estrogen induces the ascent of the entire transformation zone (Type 3 Transformation Zone), making both cytology and colposcopy ineffective [5,6]. The cervixes of these women are often small with unexplorable cervical canals, so it may also be difficult to perform a LEEP, the cone of which should be longer (15–20 mm) than it is in young women (7–10 mm) [7]. This specific group of postmenopausal patients needs more attention during treatment. Although European guidelines and the ASCCP deem the use of hysterectomy in the treatment of CIN3 unacceptable, many clinicians are more inclined to propose hysterectomy to postmenopausal women diagnosed with CIN3, considering it a definitive intervention. The literature has shown that the therapeutic efficacy of LEEPs is almost identical to that of hysterectomy with less morbidity [8]. The risk of occult carcinoma is greater in postmenopausal women, so some gynecologists resort to hysterectomy to treat CIN3. Furthermore, the literature considers hysterectomy for CIN3 a known risk factor for the subsequent development of vaginal intraepithelial neoplasia (VaIN) [9,10]. High-grade VaIN is the precursor of vaginal squamous cell carcinoma, which has an incidence 100 times lower than that of CIN [11]. According to the American Society for Colposcopy and Cervical Pathology and the College of American Pathologists, vaginal intraepithelial neoplasia (VaIN) can be classified as low-grade VaIN and high-grade VaIN [12]. The study by Castle et al. revealed that the HPV prevalence is equal in hysterectomized and non-hysterectomized women [13].

VaIN often exists in conjunction with vulvar and cervical intraepithelial lesions [14]. It is hypothesized that the hysterectomy procedure may be a cause of increased incidence of VaIN in hysterectomized women. There are few studies addressing this issue, and they show conflicting results [9,15].

This study aimed to study the incidence of high-grade VaIN in the population of women undergoing hysterectomy due to CIN3 or benign uterine pathology to illustrate the therapeutic options and follow-up.

## 2. Materials and Methods

**Figure 1:**
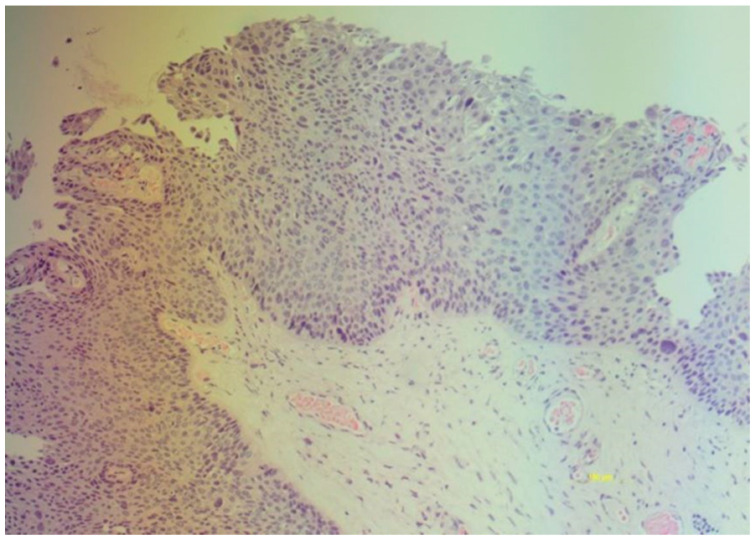
Vaginal intraepithelial neoplasia (VaIN3): full-thickness intraepithelial lesion involving more than three-thirds of the vaginal epithelium with severe dysplasia (5×).

The study was multicenter and retrospective. For the present study, we collected the medical records of women who underwent a total laparoscopic hysterectomy from January 2019 to December 2022. The second-level centers for the diagnosis and treatment of HPV lesions and cervical cancer that participated in the study were the Gynecology and Obstetrics Unit University Hospital Rodolico Department of General Surgery and Medical-Surgical Specialties of the University of Catania and the Gynecological Oncology Operational Unit, Humanitas Hospital, Catania, Italy. The medical records of 254 women screened for cervical cancer with primary HPV testing, unvaccinated and undergoing laparoscopic hysterectomy, were studied. The women in the cohort were grouped as follows: women hysterectomized due to CIN3 and women hysterectomized due to benign gynecological pathology (uterine fibroma, adenomyosis, endometrial hyperplasia). The inclusion criteria were (1) women older than 45 years; (2) without the desire to preserve their fertility; (3) with a histological diagnosis of CIN3 or benign gynecological diseases without a history of CIN (e.g., uterine fibroids, endometriosis); (4) screened with an HPV test or who had performed an HPV test with genotyping. The exclusion criteria were (1) women aged < 45 years; (2) those subjected to hysterectomy due to invasive or microinvasive neoplasia of the lower genital tract; (3) women with fertility requirements; (4) those lost to follow-up after hysterectomy; (5) those without an HPV test performed or defined; (6) those with incomplete clinical data. A histological diagnosis of high-grade VaIN was defined as the presence of cells with moderate or severe dysplasia in the context of the squamous epithelium of the vagina in the absence of invasion (Figure 1).

All women underwent laparoscopic hysterectomy. The follow-up strategy included performing a cotest and colposcopy with biopsy if necessary. The first follow-up was performed six months after the hysterectomy.

### 2.1. The HPV Test and Genotyping

Exo-endocervical cytology samples were collected in ThinPrep solution for total nucleic acid extraction for the detection and genotyping of viral DNA by means of gene amplification. This was followed by hybridization with genotype-specific probes able to identify most of the genital region HPV types. HPV testing was performed using a previously reported method [16].

The automated DNA extraction was carried out with a 1 mL sample using the NucliSENS easyMAG system (bioMérieux SA, Marcy l’Etoile, France) following the manufacturer’s HPV 1.1 protocol, with a final elution volume of 55 μL. HPV typing allowed for the identification of 11 LR genotypes (6, 11, 40, 42, 43, 44, 54, 61, 70, 72, 81) and 18 HR genotypes (16, 18, 26, 31, 33, 35, 39, 45, 51, 52, 53, 56, 58, 59, 66, 68, 73, 82).

This study conforms to the provisions of the Declaration of Helsinki, as revised in 2013. The ethics committee of the Catania University Hospital (Catania 1) was notified about the study protocol, according to the current legislation on observational studies provided by AIFA (20 March 2008), which did not request additions or changes to the protocol. According to Italian law, patient consent was not mandatory for a retrospective study [17].

Written informed consent was obtained from all the patients regarding the use of their data for scientific purposes.

### 2.2. Statistical Analysis

The statistical analysis was performed using the SPSS software package for Windows (version 15.0, SPSS, Chicago, IL, USA). Descriptive statistics are expressed as frequencies, arithmetic means, and percentages. The results are summarized in tables. The relationship between the categorical variables was assessed using Chi-square tests or Fisher’s exact tests, depending on the sample size. We compared the frequency of specific HR HPV genotypes in patients with or without VAIN lesions in the two groups considered in the study. A statistical analysis including independent χ^2^ and *t* tests was performed, with all *p* values < 0.05 considered significant.

## 3. Results

**Figure 2:**
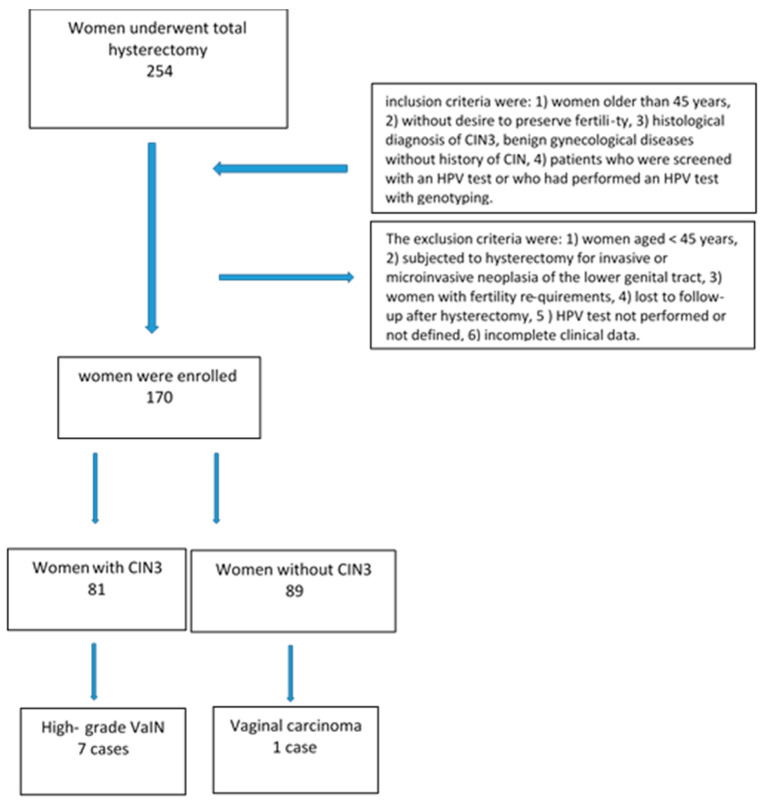
Flow-chart of study population.

Of the 254 patients, only 170 met our requirements: the study sample consisted of a group of 81 women hysterectomized due to CIN3 and 89 hysterectomized due to benign gynecological pathology (uterine fibroma, adenomyosis, endometrial hyperplasia). Figure 2 shows a flow-chart of the study population.

High-grade VaIN was found in eight patients after hysterectomy (4.7%). The incidence rates of VaIN in patients with and without a history of CIN were significant. Hysterectomized women with CIN3 showed an eight times greater risk than hysterectomized women with benign pathology of developing high-risk VaIN, with an OR = 8.32 (CI 95% 1.00–69.21) (*p* = 0.003). The risk of VaIN is low in women hysterectomized due to benign pathology, with an OR = 0.12 (CI 95% 0.01–1.00 *p* = 0.003 (Table 1)).

The rates of HR-HPV infection after hysterectomy for patients with and without a history of CIN were 12.4% (10/81) and 7.8% (7/89), respectively (Table 2).

The incidence of VaIN in patients with persistent HR-HPV infection and in those with and without a history of CIN was 70% (7/10) and 14.2% (1/7), respectively, *p* < 0.001.

In particular, seven cases of high-grade VaIN of the vaginal vault were diagnosed in women hysterectomized due to CIN3, with one invasive vaginal carcinoma in the women hysterectomized due to benign pathology.

The risk of developing VaIN is greater in women with viral persistence, with an OR = 14.00 (CI 95% 1.14–172.65) (*p* = 0.02).

The median time between primary treatment and a diagnosis of high-grade VaIN was 18 (range: 6–45) months. All cases of high-grade VaIN occurred in women with persistent HPV infection. The most frequent genotype was 16, followed by HPV31. Women with high-grade VaIN were positive for genotype 16; in one case, there was HPV16,52.

In the group of women who underwent hysterectomy due to benign uterine pathology and without a history of CIN, their average age was 54 years (range = 45–62), they had participated in screening for cervical cancer with primary HPV and 11 women were HPV-positive. After hysterectomy, only seven women were still HPV-positive, and one of them developed squamous cell carcinoma of the vagina. In the latter case, the genotyping highlighted genotype 16. Unfortunately, we did not have the cytology results of all the HPV-positive women, nor can we talk about genotyping in this group because the screening uses partial genotyping.

**Figure 3:**
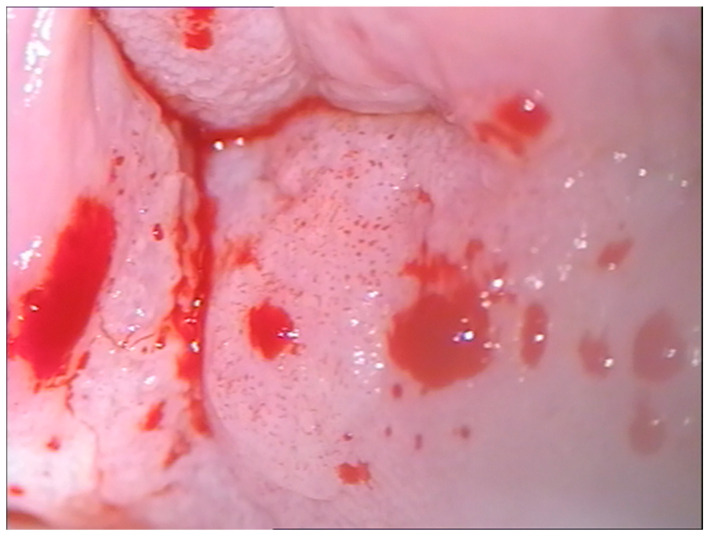
High-grade VaIN in an HPV 16-positive 62-year-old woman 20 months after undergoing hysterectomy due to CIN3.

Positive cytology for ASCUS in cases of high-grade VaIN and frank neoplasia in the case of vaginal carcinoma led us to subject the women to colposcopy. Colposcopic examination highlighted areas of a thickened, acid-positive epithelium with an irregular punctate appearance corresponding to the vaginal dome towards the posterior wall in two cases, confirmed histologically (Figure 3). In three cases, the colposcopic examination highlighted a thickened, acid-positive epithelium, with multifocal extension in two cases. Two cases of high-grade VaIN both affected the vaginal dome but were not very extensive and had the colposcopic appearance of leukoplakia. The responsible genotype was HPV16 in all cases, apart from in 1 case, where genotype 52 was associated.

**Figure 4:**
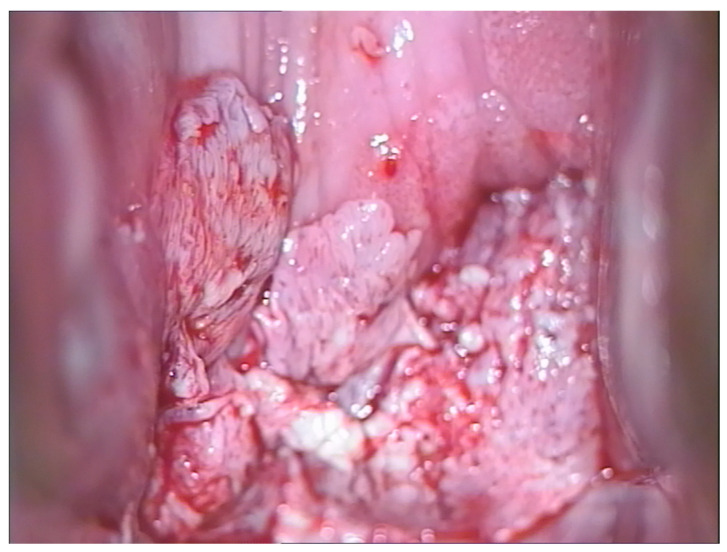
Vaginal carcinoma in an HPV 16-positive 62-year-old woman 25 months after undergoing hysterectomy due to a uterine fibroid.

Given that the lesions were high-grade and close to the closure of the vagina, it was preferred to resort to excision of the lesion with an upper vaginectomy to complete the diagnosis and exclude the presence of occult carcinoma. The colposcopic appearance of the vaginal carcinoma, 25 months after hysterectomy, was that it occupied the posterior vaginal wall towards the vaginal dome with a multipapillary appearance, easily bleeding upon probing (Figure 4). The histological examination confirmed the diagnosis, and the genotyping highlighted the presence of HPV 16. The woman underwent radiotherapy. Table 3 shows the personalized treatment according to age, histological grade and the location of the lesion.

## 4. Discussion

Previous studies demonstrate that women treated for CIN3 have an increased risk of subsequent carcinoma or high-grade anogenital preneoplastic lesions, especially vaginal cancer given the proximity of the vagina to the cervix [9,18,19,20,21,22,23,24]. The results of the present study highlight a higher rate of high-grade VaIN in HPV-positive women with a previous history of CIN3 lesions compared to women positive for HPV without previous malignancy or CIN, confirming previous studies.

This study highlights a significant correlation between the incidence of high-grade VaIN and persistent HPV infection. (*p* < 0.02), with an OR = 14.00 (95% CI 1.14–172.65). All VaIN cases were positive for the HPV 16 genotype. These data are confirmed by the study by Bryan et al., who detected HPV infection in 96% of patients diagnosed with VaIN [25]. Furthermore, a previous study revealed that the severity of VaIN was closely related to HPV 16 [26]. Therefore, HPV 16 genotyping could be a useful tool for risk stratification of patients.

Another important result highlighted by the present study is the concordance of the genotypes between high-grade VaIN and concomitant CIN3, data also confirmed by other authors [27].

All these elements suggest that it is a history of HPV-related disease of the lower genital tract and viral persistence, rather than hysterectomy itself, that should be considered risk factors for the development of high-grade VaIN. Persistent HPV infection after hysterectomy can develop vaginal, vulvar and anal dysplasia, which have similar risk factors.

The correlation between CIN3, high-grade VaIN and vulvar HSILs can be explained by the HPV-induced “field effect” theory [28].

It all begins at the level of the cervix, where the presence of a squamous–columnar junction area facilitates the oncogenic action of high-risk HPV genotypes, where a single colony of monoclonal cells is infected and transformed, giving rise to cervical CIN. Subsequently, pre-existing monoclonal cells spread the dysplastic cells throughout the epithelium of the genital mucosa, involving different sites (the cervix, vagina, vulva and anus), giving rise to multifocal disease. These lesions can occur simultaneously (synchronous lesions) or even several years after the initial cervical lesion (metachronous lesions). Cervical dysplasia usually arises years earlier than vaginal or vulvar dysplasia.

The main element that emerges is that vaginal intraepithelial neoplasia is often secondary to invasive cervical or intraepithelial neoplasia. Primary vaginal carcinoma is a rare event since the vagina, which does not have a squamous–columnar junction, is less subject to carcinogenesis than the cervix, so vaginal neoplasia is often accompanied by coexisting cervical neoplasia.

The present study shows that there is also a small risk of VaIN in women who have undergone hysterectomy due to benign uterine disease and with persistent HPV infection. Persistent HPV infection can progress undetected to vaginal cancer, as there is no follow-up program for these women.

This suggests that vaginal cytology follow-up may be warranted for all post-hysterectomy patients, but there is no consensus in the literature.

Approximately 10% of women who undergo hysterectomy due to cervical dysplasia develop vaginal dysplasia or cancer after surgery, and women who undergo a LEEP due to CIN3 may have recurrence of CIN2+; meanwhile, the onset of VaIN after hysterectomy in patients without a history of CIN is extremely rare [29,30]. In light of this, the American Society of Colposcopy and Cervical Pathology recommends that follow-up of the vagina after hysterectomy be performed only if the surgery was performed die to CIN3. Our results essentially support this strategy.

Follow-up consists of vaginal cotesting every six months for at least two years and then annually for at least ten years. Furthermore, clinical follow-up could be considered in high-risk women, such as women with HPV persistence or immunosuppressed populations, such as organ transplant recipients and HIV-positive women.

The treatment of high-grade VaIN is a very delicate topic due to the very thin vaginal wall and the close relationship it has with organs such as the urethra, bladder, rectum and Douglas space. This proximity makes standard surgical procedures and radiation therapy risky. Treatment for VaIN includes surgical excision, laser therapy and topical treatment.

Upper vaginectomy is considered the treatment of choice for high-grade VaIN in women hysterectomized for cervical cancer [31]. Surgical excision is indicated in particular when infiltration cannot be excluded; it also lends itself to a better evaluation of the margins and exclusion of invasion [32,33]. However, regardless of the treatment modality, patients with high-grade VaIN are at high risk of recurrence and are at risk of developing invasive disease [34,35].

CO_2_ laser vaporization has been used for lesions at sites other than the apex, for multifocal lesions and in sexually active young women. An indispensable condition for the use of laser therapy is the exclusion of occult invasive cancer, especially in the post-hysterectomy vaginal cuff [36]. Occult vaginal carcinomas have been reported in 6.8–18.6% of patients with HSILs [37,38]. In cases of multifocal lesions or lesions involving the lower third of the vagina, upper vaginectomy can be combined with laser vaporization [39]. Although vaginectomy is an effective operation, it cannot prevent recurrence. Furthermore, to avoid the formation of vaginal crypts, which prevent colposcopic vision and biopsy of possible lesions, some authors recommend not suturing both ends of the vaginal apex to the cardinal ligament during surgery [40].

Women with persistent HPV infection after hysterectomy due to CIN2+ who are at high risk of developing vaginal, vulvar or anal dysplasia represent the optimal target for indication of postoperative HPV vaccination. Postoperative HPV vaccination in women with HSILs may reduce the rate of lesion recurrence. There is growing evidence that vaccinating women before surgery can improve their outcomes [4,41]. A study by Sand FL et al. demonstrated that women vaccinated before surgery have a lower absolute risk of developing HSILs compared to unvaccinated women and women vaccinated after surgery. Vaccination before surgery ensures that the cervicovaginal area at the time of removal has sufficient neutralizing anti-HPV antibodies to prevent reinfection of the basal layer cells. Although these data need to be validated in patients with vaginal dysplasia, we might expect similar results [41,42].

The limitations of the present study are represented by its retrospective nature, which does not allow for a complete analysis of the data, in particular due to the lack of acquisition of data on the previous surgery and the small sample under study, with this size imposed by the rarity of the pathology. Another important limitation is the limited follow-up of the study population. A strong point is represented by the use of genotyping, which made it possible to identify women at risk after hysterectomy of preneoplastic lesions and the evidence of genotype 16. Furthermore, genotyping made it possible to highlight the concordance of the genotypes between high-grade VaIN and concomitant CIN3.

## 5. Conclusions

A total of 1% to 7% of patients undergoing hysterectomy due to CIN or cervical cancer may develop high-grade VaIN within a 2-year period [43,44].

It is recommended to routinely perform a colposcopy with possible targeted biopsy of the vaginal wall before surgery for patients undergoing hysterectomy due to CIN3 or stage IA cervical cancer. After hysterectomy, women with persistent HPV infection should undergo annual follow-up with vaginal dome cotesting.

In conclusion, the results of the present study also demonstrate the importance of the persistence of HPV 16 in the development of anogenital neoplasms in women [45].

## Figures and Tables

**Table 1:** Relationship between HG-VaIN and CIN3.

Lesion	With CIN3		Without CIN3		*p*
	Cases 81	%	Cases 89	%	
HGVaIN (n = 7)	7	8.6	0	0	0.003
Vaginal Ca (n = 1)	0	0	1	1.1	

**Table 2:** Relationship between VaIN or vaginal carcinoma and HR-HPV infection in women after hysterectomy.

HPV	With CIN		Without CIN		Value *p*
	Cases	HGVaIN	Cases	HGVaIN	
Negative	71	0	82	0	0.002
Positive	10	7	7	1	<0.001
HPV 16	8	7	1	1	<0.002
HPV 31	2	0	*	0	
Other genotypes	0	0	6	0	

**Table 3:** Personalization of treatment based on age, histological grade and location of the lesion.

Treatment	Age	HPV	Colposcopy	Location	Treatment
VaIN	45	16	thickened, acid-positive epithelium	multifocal	laser
VaIN	48	16	leucoplakia	multifocal	laser
VaIN	62	16	thickened, acid-positive epithelium with irregular punctate	vaginal dome and posterior wall	upper vaginectomy + laser
VaIN	58	16	thickened, acid-positive epithelium	vaginal dome	upper vaginectomy
VaIN	55	16	leucoplakia	vaginal dome	upper vaginectomy
VaIN	64	16	thickened, acid-positive epithelium with irregular punctate	vaginal dome and posterior wall	upper vaginectomy + laser
VaIN	60	16, 52	thickened, acid-positive epithelium	vaginal dome and posterior wall	upper vaginectomy + laser
Vaginal Carcinoma	62	16	multipapillary, easily bleeding	vaginal dome and posterior wall	radiotherapy

## Data Availability

The data are contained within the article.
